# Altered Febrile Responses in Older Adults: A Systematic Review

**DOI:** 10.1093/geroni/igaa057.722

**Published:** 2020-12-16

**Authors:** Barbara Holtzclaw

**Affiliations:** University of Oklahoma Health Sciences Center, Norman, Oklahoma, United States

## Abstract

Human and animal studies support generalizations that older adults are less able than younger adults to mount an effective febrile response. Beyond difficulties this presents for assessing signs and symptoms of infection, concern exists that older adults may lack fever’s protective immuno-stimulant benefits. Fever is a systemic physiological host response to a pyrogen resulting in release of proinflammatory cytokines that produce a regulated elevation of thermoregulatory set-point. Heat is generated, by shivering and molecular activity, and conserved, by vasomotor activity, elevating and maintaining body temperature at the higher set-point level. Because immunological, vasomotor, and kinetic activities raise body temperature, age-associated alterations have been hypothesized to explain blunted febrile responses in older adults. Purpose: A systematic review was done to 1) determine factors underlying presumed origins and alterations in older adults’ febrile responses. 2) assess for gaps and controversies in emerging research that could inform care decisions. Comparisons of disciplinary assumptions, perspectives, and cross-disciplinary interpretations sought relevance to interdisciplinary care. Methods: Search of literature databases: Medline (OVID), and CINAHL (EBSCO). PubMed, and included relevant animal and human research findings since 2000 from physiology, gerontology, immunology, infectious disease, clinical medicine, and nursing. Findings: Altered innate immunity in sepsis shows early hyper-reactive response, prolonged inflammatory activity, and fever response contributing to cardiovascular and neurological morbidity, not temperature elevation. Morbidly was attributed to disease not age. Conclusions: Hazards of blunted febrile temperatures include undetected infections and possible loss of immune benefits. Significant evidence of age-related diminished febrile temperature’s immune consequences shown with animal models.

